# Rumor spreading model considering rumor credibility, correlation and crowd classification based on personality

**DOI:** 10.1038/s41598-020-62585-9

**Published:** 2020-04-03

**Authors:** Xuelong Chen, Nan Wang

**Affiliations:** School of Economics and Management, Dalian University of Technology, Dalian, 116024 China

**Keywords:** Applied mathematics, Computational science

## Abstract

The study of rumor spreading or rumor controlling is important and necessary because rumors can cause serious negative effects on society. The process of rumor spreading is influenced by many factors. In this paper, we suggest that people with different personalities will behave differently after hearing rumors. Thus, we divide the population into two types: radical people and steady people. Furthermore, we suggest that the credibility of rumors and the correlation between rumors and people’s lives are important factors that will influence the spread of rumors. Based on these considerations, we propose the SEIsIrR model. We establish differential equations to describe the dynamics of the rumor spreading process in homogeneous and heterogeneous networks. Using the Jacobian matrix and next generation matrix, we obtain the spreading threshold of the SEIsIrR model and discuss the relationship of the spreading threshold between homogeneous networks and heterogeneous networks. We employ a real rumor dataset obtained from Twitter to verify the SEIsIrR model and perform numerical simulations in Watts-Strogatz (WS) networks and Barabasi-Albert (BA) networks to verify the obtained spreading thresholds and discuss the impacts of these factors on the rumor spreading process and the differences in the rumor spreading processes between WS networks and BA networks. The simulation results show that these factors influence the speed and range of rumor spreading.

## Introduction

With the development of the internet, rumor spreading has become easier and faster^1,2^. Social networking service (SNS) is the main platform of rumor spreading due to its numerous users and complex network structure. Everyone in social networks is both a spreader and a recipient of information. So, a rumor is easily produced and spread widely, and some rumors cause great panic in society^3,4^. For example, a rumor that nuclear leakage caused by the Fukushima nuclear accident in Japan would pollute salt was widely spread on the internet in 2011 and caused panic buying of salt. Since rumor spreading can cause serious consequences, an in-depth investigation of rumor spreading in complex social networks has significance^5^ and can help governments or managers of SNS to control rumor spreading.

The process of rumor spreading in social networks is similar to the process of epidemic spreading; thus, most studies of rumor spreading are based on the epidemic model. Daley and Kendall formalized the first rumor spreading model, namely, the DK model, in 1964; the model is based on the classic epidemic model SIR^6^. In the DK model, the crowd was divided into three groups: people who have not heard rumors (ignorants), people who spread rumors (spreaders), and people who stop spreading rumors (stiflers). With the studies of complex network theory, the effect of complex network structure on the process of rumor spreading was explored. Moreno *et al*.^7^ introduced mean field equations to characterize the rumor spreading process in complex networks. Nekovee *et al*.^8^ contrasted the rumor spreading process in a random network with the rumor spreading process in a scale-free network, the results showed that the rumor spreading model exhibits different spreading thresholds in different networks.

In addition, rumor spreading is a social contagion process, in which people’s behaviors and social environments may influence the process of rumor spreading. Thus, some researchers considered people’s behaviors and social environments in rumor spreading. Zhao *et al*. proposed a rumor spreading model that considers the forgetting mechanism, which expressed that spreaders may convert to stiflers without contacting others. These researchers also verified that the forgetting rate, which changes over time, has a significant impact on rumor spreading^9–11^. Corresponding to the forgetting mechanism, some scholars considered the remembering mechanism and yield to the SIHR model^12,13^. Wang *et al*.^14^ believed that trust between people can influence rumor spreading and proposed a rumor spreading model that considers the trust mechanism to analyze the influence of trust between people on rumor spreading. Additionally, two or more rumors would spread at the same time. Some scholars extended the classic single rumor spreading models to coupled spreading models, which consider two rumors^15–17^. Moreover, some people may refute the rumors that he or she has heard. In light of this, Zan *et al*.^18^ focused on the counterattack mechanism and analyzed the influence of networks’ self-resistance on rumor spreading. Similarly, Zhang *et al*.^19^ designed the IS_1_S_2_C_1_C_2_R_1_R_2_ propagation model to study rumor refutation in complex social networks. When people hear rumors, most people will not immediately become spreaders and contemplate whether the rumors are true. In view of this, Huo *et al*.^20^ proposed the XWYZ model. Compared with the SIR model, this model considered the new group W, which denotes people who hesitate to spread rumors. Xia *et al*.^21^ proposed the SEIR model with the hesitating mechanism and introduced the fuzziness of a rumor’s content as a parameter of this model. Hu *et al*.^22^ believed that there are three attitudes towards rumors, namely, supported, opposed and hesitant, and they verified that people who hesitate to spread rumors are positive to the spreading of rumors. Moreover, some researchers suggested that individuals’ decisions and actions depend on their states of mind and surroundings when they hear rumors^23,24^. Sahafizadeh *et al*.^25^ indicated that group propagation, where a group means a small community in which people may not directly know each other but all members can view messages sent to the group, has an impact on rumor spreading.

The differences among people and some of their attributes, such as education level and ability to identify information, may influence their decision when they hear rumors. Afassinou introduced the population’s education rate in the rumor spreading model and divided the ignorants into two parts, namely, educated ignorants and non-educated ignorant; the simulation results showed that the education rate influenced the process of rumor spreading^26^. Similarly, Wang *et al*.^27^ divided the ignorants into two parts according to their ability to identify information; the simulation results verified that the division is significance. In addition to these, Ma *et al*.^28^ applied individuals’ mastering degree of knowledge and rationality degree to depict individuals’ diverse characteristics and formed the conclusions that disseminating rumors is difficult due to the diverse characteristics of individuals. Li *et al*.^29^ discussed individuals’ sensitivity to rumor spreading based on the SIS and SIR models and concluded that lower sensitivity can inhibit the spread of rumors. Furthermore, the connection between individuals also influences rumor spreading. Cheng *et al*.^30^ developed a model with a dynamic spreading probability, which is related to the strength of individuals’ connections in a social network, and verified that the strength of connection substantially impacts the process of rumor spreading.

However, we discover that the majority of previous studies did not consider the rumor credibility in rumor spreading models. Although some scholars considered the importance and fuzziness of rumors^21,31^, these two attributes of rumor cannot properly represent the credibility of rumors. The fuzziness and importance of rumors just depicts the feature of words, whereas the credibility can fully describe the persuasiveness of rumors to people. In addition to textual content, measuring the credibility of a message requires the consideration of additional factors, such as information sources^32^. Therefore, considering the credibility is appropriate.

Although crowd classification has been introduced into rumor spreading models in some papers ^26,27^, crowd classification based on personality is a relatively new perspective. An individual’s personality describes intrinsic human traits that influence the external performance^33^. We hypothesize that a crowd can be divided into two categories based on personality. After hearing a rumor, some people are radical, that is, they easily believe what they heard. Conversely, some people are steady and calm and are likely to contemplate and seek confirmation before they decide whether the rumor is true and spread the rumor. Besides, people are more likely to focus on news that is relevant to their lives. Some studies have shown that information that is relevant to people’ lives is more easily spread^34^. For example, salt is relevant to people’s lives; the rumor that salt would be polluted by the Fukushima nuclear accident was wildly spread in China in 2011. Thus, the correlation degree between rumors and people’s lives is an important factor that influences the rumor spreading process and should be taken into account in the study of rumor spreading. However, previous studies did not adequately consider this factor.

The remainder of this paper is structured as follows: In the section titled “Rumor spreading model”, the description of our model is given. We establish differential equations to describe the dynamics of this model. The spreading thresholds of this model in homogeneous and heterogeneous networks and their relationship are also discussed in this section. In the section titled “Verification and numerical simulation”, the model is validated by real data, and numerical simulations are performed to illustrate the rumor spreading process and the impacts of different factors on the process based on our model. A brief conclusion is given in the last section.

## Rumor spreading model

In this section, a rumor spreading model, which considers rumor credibility, correlation between rumors and people’s lives and crowd classification based on personality, is proposed and referred to as the SEIsIrR model.

Based on the classic SIR model and the hesitating mechanism, we divide individuals into 5 classes according to the process of rumor spreading as follows.

- Steady ignorant. This class includes people who do not know the rumor; if they hear the rumor, they prefer to contemplate it and seek confirmation before making decisions.
- Radical ignorant. This class includes people who do not know the rumor; if they hear the rumor, they are most likely to believe it and spread it without contemplating it or seeking confirmation.
- Exposed. This class includes people who know the rumor but hesitate to believe it and do not spread it.
- Spreader. This class includes people who spread the rumor.
- Stifler. This class includes people who know the rumor but never spread it or stop spreading it.

These 5 classes are represented as Is, Ir, E, S and R, respectively. Each individual must belong to only one of the 5 classes. In addition, individuals’ classes are not fixed and can change with rumor spreading. The transition rules for the 5 classes are explained as follows:

1. Rule 1: From class Ir to class S. If a rumor is more credible and more relevant to people’s lives, people who belong to Ir are more likely to switch to class S when they hear the rumor. Thus, we assume that when an individual in class Ir comes into contact with an individual in class S and get to know the rumor, he will switch to class S with probability *γαλ*, where *γ* is the credibility of the rumor; *α* is the correlation coefficient, which depicts the degree of correlation between the rumor and people’s lives; and *λ* is the spreading probability, which depicts the probability that an ignorant individual knows the rumor via contact with an individual in class S^22^. These 3 parameters are continuous random variables that are constrained as $0\le \gamma  < 1$, $0 < \alpha \le 1$ and $0\le \lambda \le 1$. The higher *γ* is, the more credible the rumor is. When *γ* = 1, the rumor is fully credible, which means that everyone who knows the rumor will believe it; however, this situation is impossible in real life. Therefore, we limit *γ* to less than 1. The higher *α* is, the more relevant to a human’s life the rumor is. *α* = 1 implies that the rumor is closely bound to people’s lives.
2. Rule 2: From class Is to classes S and E. When an individual in class Is comes into contact with an individual in class S, he or she will switch to class S or class E with different probabilities. Since individuals in class Ir are more likely to spread rumors than individuals in class Is, we introduce the parameter *μ* ($0 < \mu \le 1$) to depict the spreading desire ratio of individuals in class Is to individuals in class Ir. The probability that individuals in class Is will switch to class S can be defined as *γαλμ*. From the previous definition, the parameter *μ* should only have a role in the process of switching from ignorants to spreaders, and individuals in class E are not spreaders because they are hesitant to spread the rumor even though they had heard it. Therefore, it is reasonable that *μ* does not act on the switching from Is to E. Conversely, individuals in class Is are calmer compared with individuals in class Ir; so, they may switch to class E with the probability *γ(*1*-γ)αλ*, where *γ* = 1 means that the rumor is fully credible, and *γ* = 0 means that the rumor is fully unreliable. None of the individuals will switch to class E in either case. When *γ* takes the median value (*γ* = 0.5), the rumor is most doubtful and the number of individuals who will switch to class E attains the maximum. In other situations, higher and lower credibility, such as when *γ* = 0.7 and *γ* = 0.3, may produce a similar hesitation degree of individuals. Therefore, we suggest that more credible and less credible rumors have a similar influence on the probability that individuals will switch from Is to E. According to reality and intuitive experience, a certain relationship between the rumor credibility and the proportion of people who hesitate should exist. We assume that the relationship is that the proportion of people who hesitate is positively correlated with *γ(1-γ)*, which will be verified by real-world data and the simulation in the section titled “Verification and numerical simulation”. Thus, the reasonable assumption that the probability that individuals in class Is will switch to class E is expressed as *γ(1-γ)αλ*.
3. Rule 3: From class E to classes S and R. Individuals in class E can switch to class S or R depending on the class which the individuals they are in contact with belong to. Individuals in class E will decide whether to spread rumors when they are influenced by others. Therefore, we assume that individuals in class E switch to class S with the probability *θ* when they are in contact with an individual in class S and switch to class R with the probability *ϕ* when they are in contact with an individual in class R.
4. Rule 4: From class S to class R. When an individual in class S comes into contact with an individual in class S, E or R, the former would switch to class R with the probability *η*_*1*_ because a spreader will realize that the rumor is not new when he or she meets another person who knows the rumor. Additionally, individuals in class S will switch to class R at the rate *η*_2_ due to the forgetting mechanism, which means that some individuals may forget the rumor in the spreading process.

**Figure 1:**
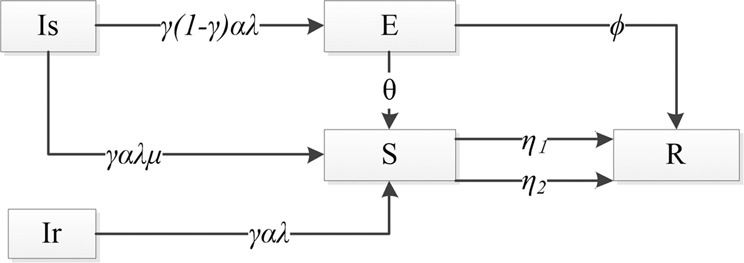
Flow diagram of rumor spreading process.

Based on transition rules 1–4, we construct a flow diagram of the rumor spreading process, as shown in Fig. 1. The meanings of the parameters of the SEIsIrR model are summarized in Table 1.

**Table 1:** Parameters of SEIsIrR model.

Parameter	Description
*α*	The correlation coefficient between rumor and people’s lives
*γ*	The credibility of a rumor
*λ*	The probability that an ignorant individual hears a rumor via contact with an individual in class S
*μ*	The spreading desire ratio
*θ*	The probability that individuals in class E will switch to class S
*ϕ*	The probability that individuals in class E will switch to class R
*η*_1_	The probability that individuals in class S will switch to class R
*η*_2_	Forgetting rate

Because the period from the birth of a rumor to its disappearance is relatively short, the change in the total number of individuals is minimal and negligible in this period. So, based on the premise that the analysis results will not be affected and to simplify the discussion, we assume that the total number of individuals is N and will remain constant during the process of rumor spreading.

Because complex networks such as social networks are usually the carriers in rumor spreading, we can discuss rumor spreading process based on the SEIsIrR model in complex networks to validate the scientificity of the SEIsIrR model. Various complex networks, such as the Watts-Strogatz (WS) network, Edös-Rényi (ER) network and Barabasi-Albert (BA) network, exist. These networks can be divided into two categories according to the degree distribution, namely, homogeneous networks and heterogeneous networks. In the next subsections, we will discuss the spreading threshold of the SEIsIrR model in homogeneous and heterogeneous networks.

### Spreading threshold of SEIsIrR model in homogeneous networks

Let ${\rho }^{Is}(t)$, ${\rho }^{Ir}(t)$, ${\rho }^{E}(t)$, ${\rho }^{S}(t)$ and ${\rho }^{R}(t)$ be the densities of individuals in class Is, Ir, E, S and R, respectively, at time t in homogeneous networks. These densities satisfy the normalization condition as follows:

1 $${\rho }^{Is}(t)+{\rho }^{Ir}(t)+{\rho }^{E}(t)+{\rho }^{S}(t)+{\rho }^{R}(t)=1$$

According to the transition rules of the SEIsIrR model, we establish the following mean-field equations to describe the evolution of ${\rho }^{Is}(t)$, ${\rho }^{Ir}(t)$, ${\rho }^{E}(t)$, ${\rho }^{S}(t)$ and ${\rho }^{R}(t)$ in the homogeneous networks:

2 $$\begin{array}{c}\frac{{\rm{d}}{\rho }^{Is}(t)}{d(t)}=-\,\overline{k}{\rho }^{Is}(t){\rho }^{S}(t)(\gamma \alpha \lambda \mu +\gamma (1-\gamma )\alpha \lambda )\\ \frac{{\rm{d}}{\rho }^{Ir}(t)}{d(t)}=-\overline{\,k}{\rho }^{Ir}(t){\rho }^{S}(t)\gamma \alpha \lambda \\ \frac{{\rm{d}}{\rho }^{E}(t)}{d(t)}=\overline{k}{\rho }^{Is}(t){\rho }^{S}(t)\gamma (1-\gamma )\alpha \lambda -\overline{k}{\rho }^{S}(t){\rho }^{E}(t)\theta -\overline{k}{\rho }^{R}(t){\rho }^{E}(t)\phi \\ \frac{{\rm{d}}{\rho }^{S}(t)}{d(t)}=\overline{k}{\rho }^{S}(t)(\mu {\rho }^{Is}(t)+{\rho }^{Ir}(t))\gamma \alpha \lambda +\overline{k}{\rho }^{S}(t){\rho }^{E}(t)\theta -\overline{k}{\rho }^{S}(t)({\rho }^{R}(t)+{\rho }^{S}(t)+{\rho }^{E}(t)){\eta }_{1}-{\rho }^{S}(t){\eta }_{2}\\ \frac{{\rm{d}}{\rho }^{R}(t)}{d(t)}=\overline{k}{\rho }^{S}(t)({\rho }^{R}(t)+{\rho }^{S}(t)+{\rho }^{E}(t)){\eta }_{1}+{\rho }^{S}(t){\eta }_{2}+\overline{k}{\rho }^{R}(t){\rho }^{E}(t)\phi \end{array}$$

where $\overline{k}$ denotes the average degree of the homogeneous networks.

The steady state of Eq. (2) is expressed as follows:

3 $$\frac{{\rm{d}}{\rho }^{Is}(t)}{d(t)}=0,\,\frac{{\rm{d}}{\rho }^{Ir}(t)}{d(t)}=0,\,\frac{{\rm{d}}{\rho }^{E}(t)}{d(t)}=0,\,\frac{{\rm{d}}{\rho }^{S}(t)}{d(t)}=0,\,\frac{{\rm{d}}{\rho }^{R}(t)}{d(t)}=0$$

Eq. (2) has a rumor-free equilibrium point E_0_ = (*x*, *y*, 0, 0, 1 − *x* − *y*), where x and y mean the densities of individuals in class Is and the densities of individuals in class Ir, respectively, when no individuals exist in class E and S. The densities of individuals in class R are represented as 1-*x*-*y* according to Eq. (1).

We employ the Jacobian matrix method to discuss the stability of Eq. (2) and obtain the rumor spreading threshold in the homogeneous networks.

The Jacobian matrix of Eq. (2) is expressed as follows:

4 $$\begin{array}{ccc}J & = & {\rm{\partial }}\left(\frac{{\rm{d}}{\rho }^{Is}(t)}{d(t)},,,\frac{{\rm{d}}{\rho }^{Ir}(t)}{d(t)},,,\frac{{\rm{d}}{\rho }^{E}(t)}{d(t)},,,\frac{{\rm{d}}{\rho }^{S}(t)}{d(t)},,,\frac{{\rm{d}}{\rho }^{R}(t)}{d(t)}\right)/{\rm{\partial }}({\rm{d}}{\rho }^{Is}(t),d{\rho }^{Ir}(t),d{\rho }^{E}(t),d{\rho }^{S}(t),d{\rho }^{R}(t))\\  & = & [\begin{array}{ccccc}-\bar{k}{\rho }^{S}(t)(\gamma \alpha \lambda \mu +\gamma (1-\gamma )\alpha \lambda ) & 0 & 0 & -\bar{k}{\rho }^{Is}(t)(\gamma \alpha \lambda \mu +\gamma (1-\gamma )\alpha \lambda ) & 0\\ 0 & -\bar{k}{\rho }^{S}(t)\gamma \alpha \lambda  & 0 & -\bar{k}{\rho }^{Ir}(t)\gamma \alpha \lambda  & 0\\ \bar{k}{\rho }^{S}(t)\gamma (1-\gamma )\alpha \lambda  & 0 & -\bar{k}{\rho }^{S}(t)\theta -\bar{k}{\rho }^{R}(t)\phi  & \bar{k}{\rho }^{Is}(t)\gamma (1-\gamma )\alpha \lambda -\bar{k}{\rho }^{E}(t)\theta  & -\bar{k}{\rho }^{E}(t)\phi \\ \bar{k}{\rho }^{S}(t)\gamma \alpha \lambda \mu  & \bar{k}{\rho }^{S}(t)\gamma \alpha \lambda  & \bar{k}{\rho }^{S}(t)\theta -\bar{k}{\rho }^{S}(t){\eta }_{1} & \bar{k}(\mu {\rho }^{Is}(t)+{\rho }^{Ir}(t))\gamma \alpha \lambda +\bar{k}{\rho }^{E}(t)\theta -\bar{k}({\rho }^{R}(t)+2{\rho }^{S}(t)+{\rho }^{E}(t)){\eta }_{1}-{\eta }_{2} & -\bar{k}{\rho }^{S}(t){\eta }_{1}\\ 0 & 0 & \bar{k}{\rho }^{S}(t){\eta }_{1}+\bar{k}{\rho }^{R}(t)\phi  & \bar{k}{\rho }^{R}(t){\eta }_{1}+\bar{k}{\rho }^{E}(t){\eta }_{1}+2\bar{k}{\rho }^{S}(t){\eta }_{1}+{\eta }_{2} & \bar{k}{\rho }^{S}(t){\eta }_{1}+\bar{k}{\rho }^{E}(t)\phi \end{array}]\end{array}$$

The Jacobian matrix at E_0_ $(x,y,0,0,1-x-y)$ is as follows:

5 $$J({E}_{0})=[\begin{array}{ccccc}0 & 0 & 0 & -\bar{k}x(\gamma \alpha \lambda \mu +\gamma (1-\gamma )\alpha \lambda ) & 0\\ 0 & 0 & 0 & -\bar{k}y\gamma \alpha \lambda  & 0\\ 0 & 0 & -\bar{k}(1-x-y)\phi  & \bar{k}x\gamma (1-\gamma )\alpha \lambda  & 0\\ 0 & 0 & 0 & \bar{k}(\mu x+y)\gamma \alpha \lambda -\bar{k}(1-x-y){\eta }_{1}-{\eta }_{2} & 0\\ 0 & 0 & \bar{k}(1-x-y)\phi  & \bar{k}(1-x-y){\eta }_{1}+{\eta }_{2} & 0\end{array}]$$

The characteristic equation of *J(E*_0_) can be obtained as follows:

6 $$\begin{array}{ccc}|J({E}_{0})\,-{\lambda }_{E}E| & = & [\begin{array}{ccccc}-{\lambda }_{{\rm{E}}} & 0 & 0 & -\bar{k}x(\gamma \alpha \lambda \mu +\gamma (1-\gamma )\alpha \lambda ) & 0\\ 0 & -{\lambda }_{{\rm{E}}} & 0 & -\bar{k}y\gamma \alpha \lambda  & 0\\ 0 & 0 & -\bar{k}(1-x-y)\phi -{\lambda }_{{\rm{E}}} & \bar{k}x\gamma (1-\gamma )\alpha \lambda  & 0\\ 0 & 0 & 0 & \bar{k}(\mu x+y)\gamma \alpha \lambda -\bar{k}(1-x-y){\eta }_{1}-{\eta }_{2}-{\lambda }_{{\rm{E}}} & 0\\ 0 & 0 & \bar{k}(1-x-y)\phi  & \bar{k}(1-x-y){\eta }_{1}+{\eta }_{2} & -{\lambda }_{{\rm{E}}}\end{array}]\\  & = & {{\lambda }_{E}}^{3}(\bar{k}(\mu x+y)\gamma \alpha \lambda -\bar{k}(1-x-y){\eta }_{1}-{\eta }_{2}-{\lambda }_{E})(\bar{k}(1-x-y)\phi +{\lambda }_{E})\end{array}$$

We can calculate the eigenvalues of the Jacobian matrix *J(E*_0_) by solving Eq. (6) and obtain the eigenvalues $(0,0,0,\overline{k}(\mu x+y)\gamma \alpha \lambda -\overline{k}(1-x-y){\eta }_{1}-{\eta }_{2},-\overline{k}(1-x-y)\phi )$. Because the first 3 eigenvalues are 0 and the last eigenvalue is non-positive, whether point E_0_ is stable depends on the fourth eigenvalue.

In similar studies, the basic reproduction number (denoted as *R*_0_) is usually employed to describe the number of secondary cases caused by a spreader in a completely ignorant population^35^. *R*_0_ < 1 represents that each spreader produces less than one new infection on average, which means that the number of spreaders does not increase and the rumor will fade. Conversely, *R*_0_ > 1 represents that the rumor will infect more people and the rumor can continuously spread in the crowd.

Based on the fourth eigenvalue, we define *R*_0_ as follows:

7 $${R}_{0}=\frac{\overline{k}(\mu x+y)\gamma \alpha \lambda }{\overline{k}(1-x-y){\eta }_{1}+{\eta }_{2}}$$

If *R*_0_ < 1, then $\overline{k}(\mu x+y)\gamma \alpha \lambda  < \overline{k}(1-x-y){\eta }_{1}+{\eta }_{2}$, that is, the fourth eigenvalue of *J(E*_0_) is negative. Since all eigenvalues are non-positive number in this case, the equilibrium point E_0_ is stable, which means that the rumor will not spread further. Conversely, if *R*_0_ > 1, the fourth eigenvalue of *J(E*_0_) is positive, and the equilibrium point E_0_ is not stable, which means that the rumor will continuously spread in the network.

### Spreading threshold of SEIsIrR model in heterogeneous networks

Nodes in heterogeneous networks have different degrees. For example, the node degree of the BA network follows a power-law distribution. For a better analysis, we divide the heterogeneous population into several homogeneous groups according to the nodes’ degree. Each group is also divided into 5 classes—Is, Ir, E, S and R—as mentioned in previous sections.

Let ${\rho }^{I{s}_{k}}(t)$, ${\rho }^{I{r}_{k}}(t)$, ${\rho }^{{E}_{k}}(t)$, ${\rho }^{{S}_{k}}(t)$ and ${\rho }^{{R}_{k}}(t)$ represent the densities of individuals with degree k in classes Is, Ir, E, S and R, respectively, at time t; they satisfy the normalization condition ${\rho }^{I{s}_{k}}(t)+{\rho }^{I{r}_{k}}(t)+{\rho }^{{E}_{k}}(t)+{\rho }^{{S}_{k}}(t)+{\rho }^{{R}_{k}}(t)=p(k)$. If we use $p(k)$ to denote the degree distribution function of a network, which means the ratio of individuals with degree k to the whole population, then we have ${\rho }^{Is}(t)=\sum _{k}{\rho }^{I{s}_{k}}(t)$. Similarly, ${\rho }^{I{r}_{k}}(t)$, ${\rho }^{{E}_{k}}(t)$, ${\rho }^{{S}_{k}}(t)$ and ${\rho }^{{R}_{k}}(t)$ can be expressed with the same pattern. The probability that nodes with degree $k$ connect to nodes with degree ${k}^{{\rm{{\prime} }}}$ can be represented as $p({k}^{{\rm{{\prime} }}}/k)$, and to simplify the discussion, we consider uncorrelated heterogeneous networks, in which $p({k}^{{\rm{{\prime} }}}/k)=k{}^{{\rm{{\prime} }}}p(k{}^{{\rm{{\prime} }}})/\bar{k}$^8^ and $\overline{k}$ is the average degree of the network. Thus, the probability that a node with degree *k* connects to a spreader node at time t is $\sum _{k{}^{{\rm{{\prime} }}}}{\rho }^{{S}_{k{}^{{\rm{{\prime} }}}}}(t)p(k{}^{{\rm{{\prime} }}}/k)$.

Based on the transition rules of the SEIsIrR model and mean-field method, we establish the mean-field equations in heterogeneous networks as follows:

8 $$\begin{array}{c}\frac{d{\rho }^{I{s}_{k}}(t)}{dt}=-\,k{\rho }^{I{s}_{k}}(t)\sum _{k{}^{{\rm{{\prime} }}}}{\rho }^{{S}_{k{}^{{\rm{{\prime} }}}}}(t)p(k{}^{{\rm{{\prime} }}}/k)(\gamma \alpha \lambda \mu +\gamma (1-\gamma )\alpha \lambda )\\ \frac{d{\rho }^{I{r}_{k}}(t)}{dt}=-k{\rho }^{I{r}_{k}}(t)\sum _{k{}^{{\rm{{\prime} }}}}{\rho }^{{S}_{k{}^{{\rm{{\prime} }}}}}(t)p(k{}^{{\rm{{\prime} }}}/k)\gamma \alpha \lambda \\ \frac{d{\rho }^{{E}_{k}}(t)}{dt}=k{\rho }^{I{s}_{k}}(t)\sum _{k{}^{{\rm{{\prime} }}}}{\rho }^{{S}_{k{}^{{\rm{{\prime} }}}}}(t)p(k{}^{{\rm{{\prime} }}}/k)\gamma (1-\gamma )\alpha \lambda -k{\rho }^{{E}_{k}}(t)\sum _{k{}^{{\rm{{\prime} }}}}{\rho }^{{S}_{k{}^{{\rm{{\prime} }}}}}(t)p(k{}^{{\rm{{\prime} }}}/k)\theta -k{\rho }^{{E}_{k}}(t)\sum _{k{}^{{\rm{{\prime} }}}}{\rho }^{{R}_{k{}^{{\rm{{\prime} }}}}}(t)p(k{}^{{\rm{{\prime} }}}/k)\phi \\ \frac{d{\rho }^{{S}_{k}}(t)}{dt}=k{\rho }^{{E}_{k}}(t)\sum _{k{}^{{\rm{{\prime} }}}}{\rho }^{{S}_{k{}^{{\rm{{\prime} }}}}}(t)p(k{}^{{\rm{{\prime} }}}/k)\theta +k({\rho }^{I{s}_{k}}(t)\mu +{\rho }^{I{r}_{k}}(t))\gamma \alpha \lambda \sum _{k{}^{{\rm{{\prime} }}}}{\rho }^{{S}_{k{\rm{{\prime} }}}}(t)p(k{\rm{{\prime} }}/k)\\ \,-k{\rho }^{{S}_{k}}(t)\sum _{k{\rm{{\prime} }}}({\rho }^{{S}_{k{\rm{{\prime} }}}}(t)+{\rho }^{{R}_{k{}^{{\rm{{\prime} }}}}}(t)+{\rho }^{{E}_{k{}^{{\rm{{\prime} }}}}}(t))p(k{}^{{\rm{{\prime} }}}/k){\eta }_{1}-{\rho }^{{S}_{k}}(t){\eta }_{2}\\ \frac{d{\rho }^{{R}_{k}}(t)}{dt}=k{\rho }^{{S}_{k}}(t)\sum _{k{}^{{\rm{{\prime} }}}}({\rho }^{{S}_{k{}^{{\rm{{\prime} }}}}}(t)+{\rho }^{{R}_{k{}^{{\rm{{\prime} }}}}}(t)+{\rho }^{{E}_{k{}^{{\rm{{\prime} }}}}}(t))p(k{}^{{\rm{{\prime} }}}/k){\eta }_{1}+{\rho }^{{S}_{k}}(t){\eta }_{2}+k{\rho }^{{E}_{k}}(t)\sum _{k{}^{{\rm{{\prime} }}}}{\rho }^{{R}_{k{}^{{\rm{{\prime} }}}}}(t)p(k{}^{{\rm{{\prime} }}}/k)\phi \end{array}$$

Eq. (8) will attain a steady state when the gradients of all densities are 0; so the steady state is represented as follows:

9 $$\frac{{\rm{d}}{\rho }^{I{s}_{k}}(t)}{d(t)}=0,\,\frac{{\rm{d}}{\rho }^{I{r}_{k}}(t)}{d(t)}=0,\,\frac{{\rm{d}}{\rho }^{{E}_{k}}(t)}{d(t)}=0,\,\frac{{\rm{d}}{\rho }^{{S}_{k}}(t)}{d(t)}=0,\,\frac{{\rm{d}}{\rho }^{{R}_{k}}(t)}{d(t)}=0$$

Eq. (8) has the rumor-free equilibrium point E_0_ = $(x{}^{{\rm{{\prime} }}},y{}^{{\rm{{\prime} }}},0,0,p(k)-x{}^{{\rm{{\prime} }}}-y{}^{{\rm{{\prime} }}})$ with the conditions ${\rho }^{{E}_{k}}(t)=0$ and ${\rho }^{{S}_{k}}(t)=0$, where $x{}^{{\rm{{\prime} }}},y{}^{{\rm{{\prime} }}}$ and $p(k)\,-\,x{}^{{\rm{{\prime} }}}\,-\,y{}^{{\rm{{\prime} }}}$ are the densities of individuals with degree k in class Is, Ir and R, respectively, when no individuals exist in class E and S.

According to the next generation matrix method^35^, we can calculate the basic reproduction number by the formula ${R}_{0}=\rho (F{V}^{-1})$, where $F{V}^{-1}$ is referred to as the next generation matrix and $\rho (X)$ is the spectral radius of *X*. *F* and *V* can be instantiated by the differential of ${\mathcal F}$ and ${\mathscr{V}}$ respectively, at *E*_0_, where ${\mathcal F}$ denotes the rate of production of new infections, and ${\mathscr{V}}$ denotes the rate of change in the 5 classes. ${\mathscr{V}}$ can be obtained by the formula ${\mathscr{V}}={{\mathscr{V}}}_{-}-{{\mathscr{V}}}_{+}$, where ${{\mathscr{V}}}_{-}$ denotes the outflow rate of individuals in the 5 classes and ${{\mathscr{V}}}_{+}$ denotes the inflow rate of individuals in the 5 classes.

In this paper, individuals who switch from class E to class S are not considered to be new infections because individuals in class E and S have already heard rumors, which means that they are infected individuals^35^. Based on the next generation matrix and Eq. (8), we have the following:

10 $${\mathscr{F}}=[\begin{array}{c}k{\rho }^{I{s}_{k}}(t)\sum _{k{}^{{\rm{{\prime} }}}}{\rho }^{{S}_{k{}^{{\rm{{\prime} }}}}}(t)p(k{}^{{\rm{{\prime} }}}/k)\gamma (1-\gamma )\alpha \lambda \\ k({\rho }^{I{s}_{k}}(t)\mu +{\rho }^{I{r}_{k}}(t))\sum _{k{}^{{\rm{{\prime} }}}}{\rho }^{{S}_{k{}^{{\rm{{\prime} }}}}}(t)p(k{}^{{\rm{{\prime} }}}/k)\gamma \alpha \lambda \end{array}]$$

11 $${\mathscr{V}}=[\begin{array}{c}k{\rho }^{{E}_{k}}(t)\sum _{k{}^{{\rm{{\prime} }}}}{\rho }^{{S}_{k{}^{{\rm{{\prime} }}}}}(t)p(k{}^{{\rm{{\prime} }}}/k)\theta +k{\rho }^{{E}_{k}}(t)\sum _{k{}^{{\rm{{\prime} }}}}{\rho }^{{R}_{k{}^{{\rm{{\prime} }}}}}(t)p(k{}^{{\rm{{\prime} }}}/k)\phi \\ k{\rho }^{{S}_{k}}(t)\sum _{k{}^{{\rm{{\prime} }}}}({\rho }^{{S}_{k{}^{{\rm{{\prime} }}}}}(t)+{\rho }^{{E}_{k{}^{{\rm{{\prime} }}}}}(t)+{\rho }^{{R}_{k{}^{{\rm{{\prime} }}}}}(t))p(k{}^{{\rm{{\prime} }}}/k){\eta }_{1}+{\rho }^{{S}_{k}}(t){\eta }_{2}-k{\rho }^{{E}_{k}}(t)\sum _{k{}^{{\rm{{\prime} }}}}{\rho }^{{S}_{k{}^{{\rm{{\prime} }}}}}(t)p(k{}^{{\rm{{\prime} }}}/k)\theta \end{array}]$$

By differentiating Eq. (10) and Eq. (11) at E_0_ $(x{\rm{{\prime} }},y{\rm{{\prime} }},0,0,p(k)-x{\rm{{\prime} }}-y{\rm{{\prime} }})$, we can obtain the following:

12 $$F=[\begin{array}{cc}0 & kx{}^{{\rm{{\prime} }}}\sum _{k{}^{{\rm{{\prime} }}}}p(k{}^{{\rm{{\prime} }}}/k)\gamma (1-\gamma )\alpha \lambda \\ 0 & k(x{}^{{\rm{{\prime} }}}\mu +y{}^{{\rm{{\prime} }}})\sum _{k{}^{{\rm{{\prime} }}}}p(k{}^{{\rm{{\prime} }}}/k)\gamma \alpha \lambda \end{array}]$$

13 $$V=[\begin{array}{cc}k\sum _{k{}^{{\rm{{\prime} }}}}(p(k)-x{}^{{\rm{{\prime} }}}-y{}^{{\rm{{\prime} }}})p(k{}^{{\rm{{\prime} }}}/k)\phi  & 0\\ 0 & k\sum _{k{}^{{\rm{{\prime} }}}}(p(k)-x{}^{{\rm{{\prime} }}}-y{}^{{\rm{{\prime} }}})p(k{}^{{\rm{{\prime} }}}/k){\eta }_{1}+{\eta }_{2}\end{array}]$$

We obtain the inverse of V as follows:

14 $${V}^{-1}=\left[\begin{array}{cc}\frac{1}{k\sum _{k{}^{{\rm{{\prime} }}}}(p(k)-x{}^{{\rm{{\prime} }}}-y{}^{{\rm{{\prime} }}})p(k{}^{{\rm{{\prime} }}}/k)\phi } & 0\\ 0 & \frac{1}{k\sum _{k{}^{{\rm{{\prime} }}}}(p(k)-x{}^{{\rm{{\prime} }}}-y{}^{{\rm{{\prime} }}})p(k{}^{{\rm{{\prime} }}}/k){\eta }_{1}+{\eta }_{2}}\end{array}\right]$$

The basic reproduction number for Eq. (8) is the spectral radius of the next generation matrix $F{V}^{-1}$ ^35^, and the basic reproduction number for the heterogeneous population is the sum of the basic reproduction number for homogeneous groups with different degrees. Therefore, we can obtain *R*_0_ for the entire population as follows:

15 $$\begin{array}{c}{R}_{0}=\sum _{k}\rho (F{V}^{-1})\\ =\sum _{k}\frac{k(x{}^{{\rm{{\prime} }}}\mu +y{}^{{\rm{{\prime} }}})\sum _{k{}^{{\rm{{\prime} }}}}p(k{}^{{\rm{{\prime} }}}/k)\gamma \alpha \lambda }{k\sum _{k{}^{{\rm{{\prime} }}}}(p(k)-x{}^{{\rm{{\prime} }}}-y{}^{{\rm{{\prime} }}})p(k{}^{{\rm{{\prime} }}}/k){\eta }_{1}+{\eta }_{2}}=\sum _{k}\frac{k(x{}^{{\rm{{\prime} }}}\mu +y{}^{{\rm{{\prime} }}})\gamma \alpha \lambda }{k\sum _{k{}^{{\rm{{\prime} }}}}(p(k)-x{}^{{\rm{{\prime} }}}-y{}^{{\rm{{\prime} }}})p(k{}^{{\rm{{\prime} }}}/k){\eta }_{1}+{\eta }_{2}}\\ =\sum _{k}\frac{k(x\mu +y)\gamma \alpha \lambda p(k)}{k\sum _{k{}^{{\rm{{\prime} }}}}(1-x-y)p(k)p(k{}^{{\rm{{\prime} }}}/k){\eta }_{1}+{\eta }_{2}}=\sum _{k}\frac{k(x\mu +y)\gamma \alpha \lambda p(k)}{kp(k)(1-x-y){\eta }_{1}+{\eta }_{2}}\end{array}$$

where $x$ and $y$ denote the densities of individuals in class Is and class Ir for the whole population, $x=\frac{x{}^{{\rm{{\prime} }}}}{p(k)}$ and $y=\frac{y{}^{{\rm{{\prime} }}}}{p(k)}$. Here, we use $p(k)$ to convert *x*′ and *y*′ into *x* and *y*, so that the *R*_0_ of homogeneous network and heterogeneous network can be further compared with each other.

### Relationship between the spreading thresholds in homogeneous networks and heterogeneous networks

In the above analysis, we have obtained the spreading thresholds of the SEIsIrR model in the homogeneous networks and the heterogeneous networks, which are expressed in Eq. (7) and Eq. (15). Comparing these two equations, we can obtain ${R}_{0}=\frac{\overline{k}(x\mu +y)\gamma \alpha \lambda }{{\eta }_{2}}$ in both the homogeneous networks and the heterogeneous networks as *η*_1_ approaches 0. Therefore, the results concluded that the basic reproduction number in the heterogeneous networks is equivalent to that in the homogeneous networks when *η*_1_ approaches 0. The basic reproduction number depends on the average degree of a network rather than the degree distribution of a network when the rate of individuals in class S who switch to class R approaches 0.

To further analyze the numerical relationship between *R*_0_ in the homogeneous network and *R*_0_ in the heterogeneous network, we can obtain other forms of *R*_0_ in the homogeneous networks and heterogeneous networks by further conversion of Eq. (7) and Eq. (15), as shown in Eq. (16) and Eq. (17), respectively:

16 $${R}_{0}=\frac{\overline{k}(x\mu +y)\gamma \alpha \lambda }{\overline{k}(1-x-y){\eta }_{1}+{\eta }_{2}}=\frac{{a}_{1}}{{a}_{2}}-{\eta }_{2}\frac{{a}_{1}}{{a}_{2}}\frac{1}{\overline{k}+{\eta }_{2}}$$

17 $${R}_{0}=\sum _{k}\frac{k(x\mu +y)\gamma \alpha \lambda p(k)}{kp(k)(1-x-y){\eta }_{1}+{\eta }_{2}}=\frac{{a}_{1}}{{a}_{2}}-{\eta }_{2}\frac{{a}_{1}}{{a}_{2}}\sum _{k}\frac{1}{kp(k)+{\eta }_{2}}$$

where

$$\begin{array}{c}a1=(x\mu +y)\gamma \alpha \lambda \\ a2=(1-x-y){\eta }_{1}\end{array}$$

To make *R*_0_ in the homogeneous networks comparable to *R*_0_ in the heterogeneous networks, we set the average degree $\overline{k}$ of the two networks to the same value. Because $\overline{k}=\sum _{k}kp(k)$, we can obtain $kp(k) < \overline{k}$. Comparing Eq. (16) and Eq. (17), *R*_0_ in the heterogeneous networks is less than *R*_0_ in the homogeneous networks. This result shows that the spreading threshold of heterogeneous networks is less than that of homogeneous networks when the parameters and the average degree of networks are equivalent in both networks. In other words, rumors spread more widely in the homogeneous networks.

## Verification and numerical simulation

In this section, we verify the SEIsIrR model by using a real rumor dataset of Twitter named Pheme and numerical simulations. Additionally, the impacts of different parameters on the rumor spreading process and the differences between the rumor spreading processes in a homogeneous network and those in a heterogeneous network are also discussed.

The WS network is a typical homogeneous network. Compared with the ER network, the degree distribution of the WS network is more concentrated, which is more consistent with the nature of a homogeneous network. The WS network is closer to social networks in reality. As to heterogeneous network, BA is the typical representative, which has been extensively recognized. Therefore, we simulate the process of rumor spreading based on the WS network and BA network in numerical simulation verification.

### Model verification

#### Verification by actual data

The Pheme dataset is used to verify the rumor spreading model. First, we obtain categories that correspond to the classes in our model by classifying the tweets in the Pheme dataset according to their properties. Second, we adjust the parameters in our model to make the curve obtained by the model simulation best fit the curve generated by the data from the Pheme dataset. Last, we analyze the reasons for the slight difference between the two curves.

The Pheme dataset contains 4824 tweets associated with 9 different events on Twitter^36^. We choose two events: Charlie Hebdo and Sydney Siege. The reason for choosing these two events is that they contain the most tweets, and the larger the number of tweets is, the more obvious the transmission characteristics are.

The Pheme dataset has two types of tweets for each event: source tweet and reply tweet. A source tweet is an initial tweet, and a reply tweet is a response to a source tweet. Table 2 illustrates some tweets’ properties and possible values that we use to classify tweets. The property “support” denotes the attitudes of source tweets. The property “response-vs-source” denotes the attitudes of reply tweets to source tweets. The property “certainty” denotes the degree of certainty with which the tweets are expressed.

**Table 2:** Properties of tweets and their possible values.

	properties	values
source tweets	support	supporting, denying, underspecified
reply tweets	response-vs-source	agreed, disagreed, appeal for more information
	certainty	certain, somewhat-certain, uncertain, underspecified

The data in the Pheme dataset is the reflection of all individuals and their behaviors involved in rumor spreading over a certain period of time. In our model, Is and Ir denote people who are not currently involved in rumor spreading, and R demotes people who know the rumor but never spread it or stop spreading it. So, capturing the changes in the number of individuals in classes Is, Ir and R is difficult in the Pheme dataset. Depending on the tweets’ properties, we label some tweets in the Pheme dataset as rumor tweets or hesitating tweets that correspond to class S and E in our model.

For example, if the value of “support” property is supporting, the source tweet is a rumor tweet and the source tweet’s reply tweet is a rumor tweet if the value of “response-vs-source” is agreed. Despite the value of the other property, if the value of “certainty” is uncertain, the tweet is judged to be a hesitating tweet. Although antirumors do not belong to the content of this paper, a reply tweet to an antirumor tweet is judged to be a rumor tweet if the reply tweet has a “response-vs-source” property value of “disagreed”. So, the antirumor tweets in the Pheme dataset are considered in the labeling process. The process of labeling tweets can be summarized as the following pseudo code.

if x is source tweet:

    if x.support == supporting:

        x = rumor tweet

    else if x.support == denying:

        x = antirumor tweet

else if x is reply tweet:

    if x.reponse-vs-source == appeal for more information or x.certainty == uncertain

        x = hesitating tweet

       else if x.source == rumor tweet:

        if x.reponse-vs-source == agreed:

            x = rumor tweet

        if x.reponse-vs-source == disagreed:

            x = antirumor tweet

    else if x.source == antirumor tweet:

        if x.reponse-vs-source == agreed:

            x = antirumor tweet

        if x.reponse-vs-source == disagreed:

            x = rumor tweet

We can label tweets according to their properties; however, specific parameters’ values, such as *γ*, in the SEIsIrR model cannot be obtained from the Pheme dataset. Therefore, we adjust the parameters’ values to obtain a model that closely reflects the real data, and the parameters’ values that make the model derived curve to best fit the real data derived curve would be the best values. We perform multiple simulations with different parameters’ values, which are iteratively changed from 0.1 to 1, and obtain the specific parameters’ values in Table 3, which can render the model derived curve that resembles the real data derived curve. In addition to the parameter adjustment, the initial population classification should also be set for the verification process. Because the ratio of the quantity of radical ignorant to the quantity of steady ignorant is hardly available in the Pheme dataset, the ratio is set to 6:4 after many experiments.

**Table 3:** Parameters of model for Charlie Hebdo and Sydney Siege rumor.

parameters	Charlie Hebdo	Sydney Siege
*γ*	0.6	0.5
*α*	0.65	0.5
*δ*	0.6	0.5
*μ*	0.08	0.1
*φ*	0.7	0.3
*η*_1_	0.65	0.2
*θ*	0.15	0.2
*η*_2_	0.18	0.2

**Figure 2:**
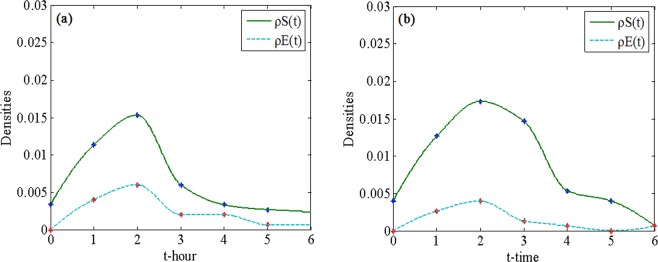
Spreading process of Charlie Hebdo rumor on (**a**) Pheme dataset and (**b**) BA network.

**Figure 3:**
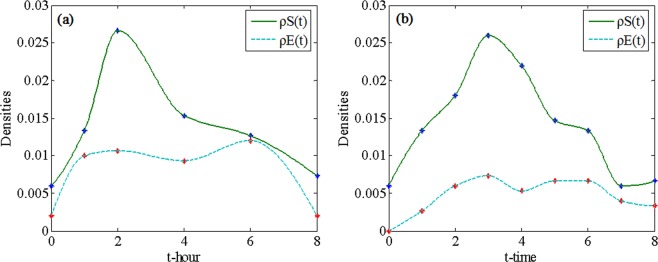
Spreading process of Sydney Siege rumor on (**a**) Pheme dataset and (**b**) BA network.

For the Charlie Hebdo event, Fig. 2(a) shows how the number of rumor tweets and hesitating tweets changed in the first 6 hours on Pheme dataset. The reason that we choose the first 6 hours is that this duration is considered a complete rumor spreading process. Although secondary transmission may occur after a few hours, it may constitute another spreading process. Fig. 2(b) shows the simulated evolution of densities of individuals in classes E and S in the BA network. Similarly, we can observe how the number of rumor tweets and hesitating tweets changed in the Pheme dataset and the simulated dynamics of the BA network for the Sydney Siege event in Fig. 3. The BA network is chosen for the simulation because it is closer to real social networks. A comparison of Fig. 2(a,b) or a comparison of Fig. 3(a,b) reveals that the dynamics of the real-world dataset and our model are similar. The slight difference is attributed to the network structure, that is, the BA network is not exactly the same as the real-world network in terms of the network structure.

#### Verification by numerical simulations

Assume that a WS network consists of 10 000 nodes, which means that a rumor is spreading among 10000 individuals, and the average degree of the network is 10. For the BA network, we set the number of edges that connect to each new node to 5, which means that each new node chooses 5 nodes to connect. The node degree of the BA network follows a power-law distribution. For ease of comparison, the number of nodes and the average degree of the BA network are set to 10 000 and 10, respectively, which is consistent with the size and average degree of the WS network. Based on these settings of the network structure parameter, we apply the NetworkX package in Python to generate the initial WS and BA networks. In the simulation process, the value of parameter t represents one tenth of the number of iterations of the simulation, and the 5 classes are dynamically updated in every iteration according to the transition rules among the 5 classes. Additionally, we assume that at the initial time (t = 0), the number of individuals in class S is 10, and the remaining individuals are people who have not heard the rumor, namely, belong to class Ir or Is.

**Figure 4:**
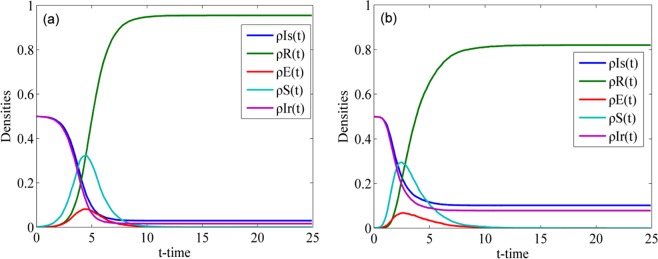
Rumor spreading process in (**a**) WS network and (**b**) BA network.

Fig. 4 displays the process of rumor spreading with the parameter settings *γ* = 0.7, *α* = 0.8, *λ* = 0.7, *μ* = 0.5, *ϕ* = 0.1, *θ* = 0.1, *η*_1_ = 0.1 and *η*_2_ = 0.l in the WS and BA networks. In both networks, the densities of the individuals in class S increase until they attain their peak values and then gradually decrease. The densities of the individuals in class E have the same trend as the densities of the individuals in class S. The densities of individuals in classes Is and Ir always decrease until a steady state is attained. When the rumor spreading process attains a steady state, individuals only exist in classes Is, Ir and R, and the densities of the individuals in class R attain their maximum values. These phenomena are in accord with the analysis conclusions of Eq. (2) and Eq. (8).

The final value of ${\rho }^{R}(t)$ in the WS network is greater than that in the BA network but the rising ^s^peed of the curve ${\rho }^{S}(t)$ in the BA network is faster than that in the WS network. The final value of ${\rho }^{R}(t)$ can be used to denote the final scale of rumor spreading because it is the density of individuals who have ever known the rumor throughout the rumor lifecycle. Thus, we can conclude that the final scale of rumor spreading in the WS network is larger than that in the BA network. However, the rumor spreading speed at the beginning stage in the BA network is faster than that of the WS network. These conclusions confirm that the central nodes, which have a large number of connected edges in the BA network, accelerate the spreading of rumors and restrain further spreading of rumors once they switch to the R class. Therefore, the final scale of rumor spreading in the BA network is smaller than that in the WS network.

**Figure 5:**
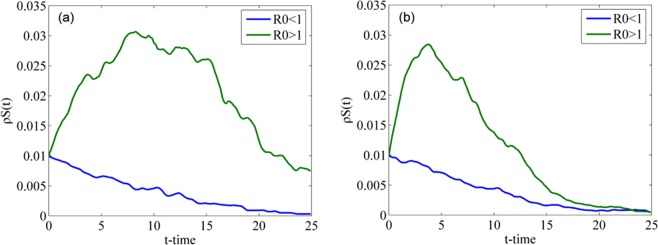
Densities of individuals in class S over time when *R*_0_ < 1 and *R*_0_ > 1 in (**a**) WS network and (**b**) BA network.

To verify Eq. (7) and Eq. (15) and explore the influence of the value of *R*_0_ on rumor spreading, we set ${\rho }^{S}(0)=100$ to highlight the change in ${\rho }^{S}(t)$ (${\rho }^{S}(0)$ is set to 100 only for Fig. 5) and simulate the evolution of the densities of individuals in class S when *R*_0_ < 1 and *R*_0_ > 1. Fig. 5(a) shows two cases for the WS network: when *R*_0_ < 1 and when *R*_0_ > 1. In the case of *R*_0_ < 1, we set the spreading probability *λ* = 0.06 and obtain *R*_0_ = 0.81 based on Eq. (7). According to the definition of *R*_0_, the density of individuals in class S decreases when *R*_0_ < 1, which means that the rumor does not spread further. In the case of *R*_0_ > 1, we set the spreading probability *λ* = 0.3. By Eq. (7), we obtain *R*_0_ = 4.05, which is greater than 1. This finding means that on average every spreader can infect more than one ignorant when *R*_0_ > 1; thus, the rumor can be propagated. Fig. 5(b) shows two cases in the BA network: when *R*_0_ < 1 and when *R*_0_ > 1. Similar to the two cases in the WS network, if *R*_0_ < 1, the curve falls until the density of individuals in class S approaches 0, which means that rumors do not spread further. Conversely, if *R*_0_ > 1, the curve rises and then gradually falls, which means that more individuals are infected by rumors.

### Discussion on the impacts of parameters

To maintain the consistency of the analysis process, the simulation process in this subsection adopts the network generation parameters in the sub-subsection titled “Verification by numerical simulations”.

#### Impact of credibility of rumor

**Figure 6:**
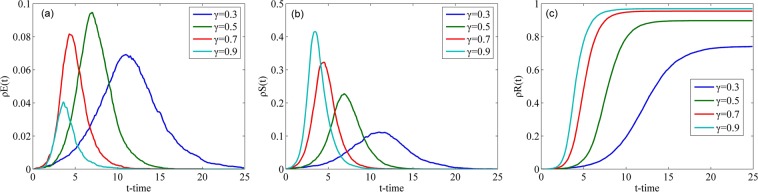
Densities of individuals in classes E, S and R over time with different *γ* in the WS network.

Fig. 6 shows the evolution of the densities of individuals in classes E, S and R over time with different *γ* in the WS network. As shown in Fig. 6(a), as *γ* increases, the peak value of ${\rho }^{E}(t)$ decreases when *γ* > 0.5. Conversely, the peak value of ${\rho }^{E}(t)$ increases with an increase in *γ* when *γ* < 0.5. When a rumor is fully credible or completely untrustworthy, no hesitation will occur. When *γ* is approaching 0.5, the rumor becomes more confusing and more people will doubt the rumor. Therefore, the maximum value of ${\rho }^{E}(t)$ will appear when *γ* = 0.5. As shown in Fig. 6(b), the peak value of ${\rho }^{S}(t)$ is positively correlated with *γ*. With an increase in *γ*, the time for ${\rho }^{S}(t)$ to attain its peak decreases. As shown in Fig. 6(c), the larger the value of *γ* is, the larger the final value of ${\rho }^{R}(t)$ is and the shorter the time to attain a steady state is. Thus, we can conclude that the increase in *γ* will cause an increase in the speed and the final scale of rumor spreading.

**Figure 7:**
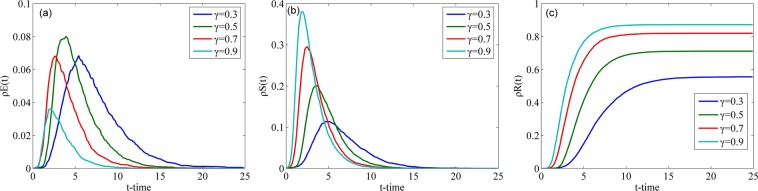
Densities of individuals in classes E, S and R over time with different *γ* in the BA network.

Fig. 7 illustrates the changes in the densities of individuals in classes E, S and R over time with different *γ* in the BA network. Compared with Fig. 6, similar changes occur in the WS network, that is, the maximum densities of individuals in class E appears when *γ* is 0.5 (refer to Figs. 6(a) and 7(a)). However, the curves with different *γ* in the BA network are closer to each other than those in the WS network (refer to Figs. 6(a,b) and 7(a,b), which means the differences in the time to attain their peak values with different *γ* in the BA network are smaller than those in the WS network. Central nodes in the BA network accelerate the spread of rumors and reduce the influence of the increase in *γ* on rumor spreading.

#### Impact of crowd classification

**Figure 8:**
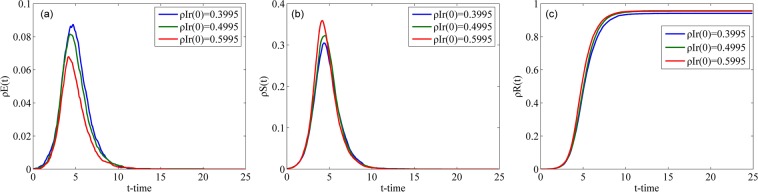
Densities of individuals in classes E, S and R over time with different ${\rho }^{Ir}(0)$ in the WS network.

Fig. 8 describes the changes in the densities of individuals in classes E, S and R over time with different ${\rho }^{Ir}(0)$ in the WS network, where ${\rho }^{Ir}(0)$ represents the initial proportion of individuals in class Ir. Since classes E and R have no individuals at the initial time, that is, ${\rho }^{E}(0)={\rho }^{R}(0)=0$, ${\rho }^{Is}(0)+{\rho }^{Ir}(0)+{\rho }^{S}(0)=1$. Because ${\rho }^{S}(0)=0.001$, the sum of ${\rho }^{Is}(0)$ and ${\rho }^{Ir}(0)$ is a definite value, that is, given the value of ${\rho }^{Ir}(0)$, the initial population classification is determined. ${\rho }^{Ir}(0)$ is set to 0.3995, 0.4995 and 0.5995, which means that the initial number of individuals in class Ir is less than, equal to and greater than the initial number of individuals in class Is. As shown in Fig. 8, the smaller ${\rho }^{Ir}(0)$ is, the higher the peak of ${\rho }^{E}(t)$ is. Conversely, the larger ${\rho }^{Ir}(0)$ is, the higher the peak of ${\rho }^{S}(t)$ is and the larger the final value of ${\rho }^{R}(t)$ is. According to these phenomena, if the number radical people in a crowd increases, the speed of rumor spreading increases, the number of spreaders increases, and the number of people who hesitate to spread rumors decreases.

**Figure 9:**
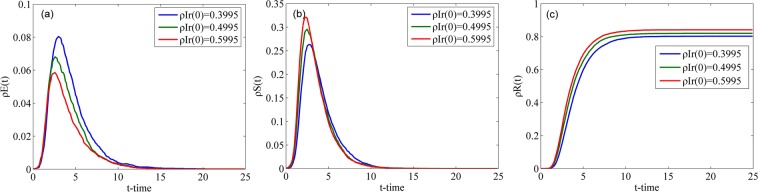
Densities of individuals in classes E, S and R over time with different ${\rho }^{Ir}(0)$ in the BA network.

Fig. 9 describes the changes in the densities of individuals in classes E, S and R over time with different ${\rho }^{Ir}(0)$ in the BA network. Compared with Fig. 8, we can conclude that the change trends of ${\rho }^{E}(t)$, ${\rho }^{S}(t)$ and ${\rho }^{R}(t)$ are identical between the WS network and the BA network. However, the peak values of ${\rho }^{E}(t)$ and ${\rho }^{S}(t)$ in the WS network are greater than those in the BA network, and the time to reach their peak values in the BA network is a slightly less. The reason for these differences is the existence of central nodes in the BA network.

#### Impact of correlation coefficient

**Figure 10:**
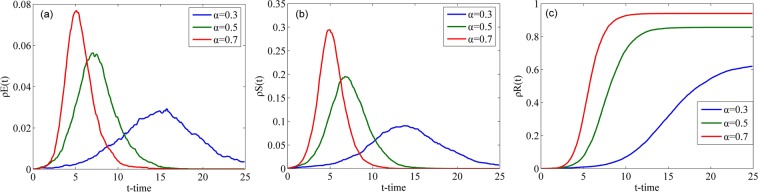
Densities of individuals in classes E, S and R over time with different *α* in the WS network.

Fig. 10 illustrates how the densities of individuals in classes E, S and R change over time with different *α* in the WS network. As shown in Fig. 10, with an increase in parameter *α*, both the peak values of ${\rho }^{E}(t)$ and ${\rho }^{S}(t)$ and the final value of ${\rho }^{R}(t)$ increase, and the time to attain their steady states decreases. The higher the correlation coefficient between a rumor and people’s lives is, the larger the spreading scale of the rumor is and the faster the speed of rumor spreading is. This conclusion accords with the reality.

**Figure 11:**
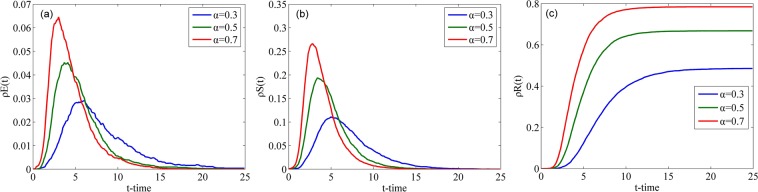
Densities of individuals in classes E, S and R over time with different *α* in the BA network.

Fig. 11 describes the changes in the densities of individuals in classes E, S and R over time with different *α* in the BA network. Compared with Fig. 10, the total trend of the curves in Fig. 11 are identical to that in Fig. 10. However, each of the curves in Fig. 10(a,b) is symmetrical about its peak value, and each of the curves in Fig. 11(a,b) is asymmetrical. The nodes in the WS network have almost the same number of connecting edges, and the increasing processes of the values of ${\rho }^{E}(t)$ and ${\rho }^{S}(t)$ are similar to their decreasing processes.

#### Comprehensive impact of credibility of a rumor and correlation coefficient

**Figure 12:**
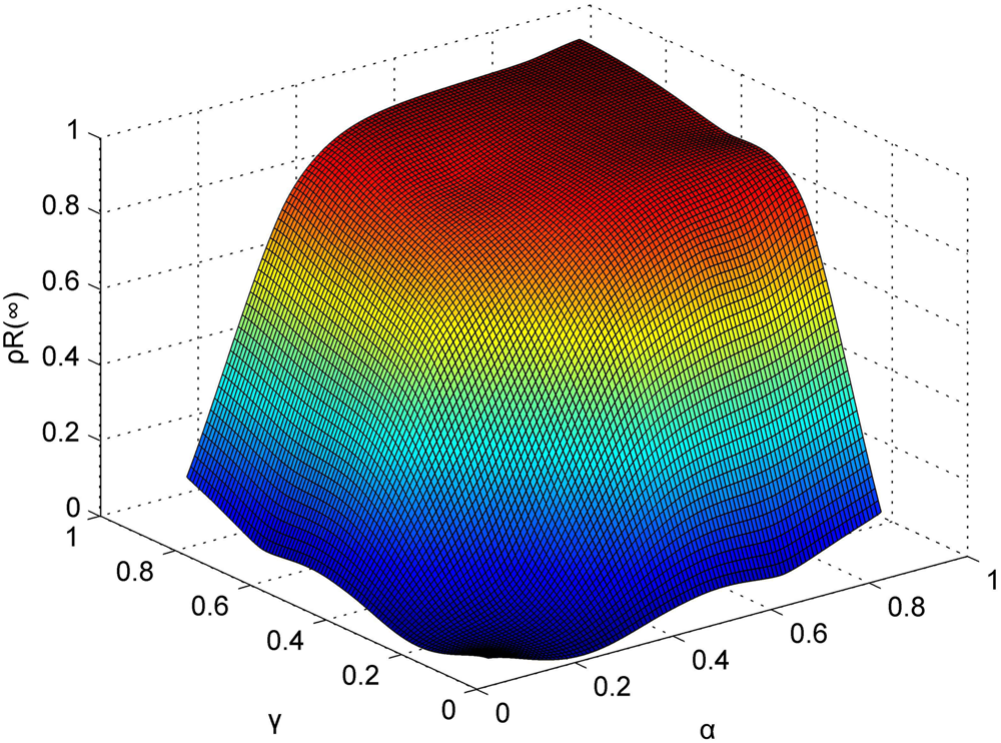
Final densities of individuals in class R with different *γ* and *α*.

In this part, we investigate the comprehensive impact of the credibility of a rumor and the correlation coefficient between a rumor and people’s lives on rumor spreading to further understand the rumor spreading mechanism in the SEIsIrR model. The relationship between the final densities of individuals in class R, which is denoted as ${\rho }^{R}(\infty )$ and the two parameters *γ* and *α*, is shown in Fig. 12, where we set $0.1\le {\rm{\gamma }}\le 0.9$ and $0.1\le {\rm{\alpha }}\le 0.9$. The value of ${\rho }^{R}(\infty )$ will be very close to 0 or 1 when these two parameters are less than 0.1 or greater than 0.9. As shown in Fig. 12, ${\rho }^{R}(\infty )$ attains its maximum value of 0.98 when *γ* = 0.9 and *α* = 0.9. When the values of *γ* and *α* are greater than 0.5, the curve slowly falls with a decrease in *γ* and *α*. However, when *γ* and *α* are less than 0.5, the curve rapidly falls with a decrease in *γ* and *α*. Therefore, we can obtain the conclusion that ${\rho }^{R}(\infty )$ is more sensitive to *γ* and *α* when *γ* and *α* is less than 0.5. Comparing the trend of a surface on the *α* coordinate axes with that on the *γ* coordinate axes, the similar trends indicate that the impacts of *γ* and *α* on ${\rho }^{R}(\infty )$ are similar. Small increases in *γ* or *α* cause large changes in the final spreading scale of a rumor unless the value of ${\rho }^{R}(\infty )$ approaches 1. This finding suggests that the credibility of a rumor and the correlation coefficient between a rumor and people’s lives are important factors of rumor spreading.

## Conclusion

Inspired by real life and based on previous studies, first, we extended the classic SIR model to propose SEIsIrR model considering the crowd classification based on personality, the correlation degree between rumors and people’s lives and the rumor credibility in this paper. Second, we applied the Jacobian matrix and next generation matrix to analyze the spreading threshold of SEIsIrR model in homogeneous and heterogeneous networks and discussed the relationship between the spreading threshold in homogeneous networks and that in a heterogeneous networks. Last, we validated the model by real data and numerical simulation, simulated the process of rumor spreading using different parameters and explored the impacts of crowd classification, rumor credibility and correlation coefficient between rumors and people’s lives on the rumor spreading process in the WS and BA networks by using Python.

We obtained significant results:

1. The simulation results showed that when the credibility is closer to its intermediate value, more people will hesitate to spread rumors, and the higher the credibility of a rumor is, the larger the final scale of rumor spreading is. If a social network has more radical people, the speed and final scale of rumor spreading is faster and larger. The higher the correlation degree between a rumor and people’s lives is, the larger the spreading scale of a rumor is and the faster the speed of rumor spreading is.
2. In the same conditions, the speed of rumor spreading in heterogeneous networks is faster than that in homogeneous networks. However, the scale of rumor spreading in heterogeneous networks is smaller than that in homogeneous networks. The central nodes in heterogeneous networks generates these differences.

In the future, we will consider the following two possible extensions. First, the further fine-grained population classification from the perspective of personality is closer to the social reality, so we might get the probability distribution of people with different personalities by analyzing real-world data to extend the SEIsIrR model and make it more practical. Second, the credibility and the correlation degree of a rumor varies for different people. So, we may further refine the characteristics of the correlation and rumor credibility instead of just characterizing them by single parameters.

## Data Availability

Correspondence and requests for materials should be addressed to X.C.

## References

[1] Qiu X, Zhao L, Wang J, Wang X, Wang Q (2016). Effects of time-dependent diffusion behaviors on the rumor spreading in social networks. Phys. Lett. A.

[2] Centola D (2010). The Spread of Behavior in an Online Social Network Experiment. Science.

[3] Wang, Q., Yang, X. & Xi, W. Effects of group arguments on rumor belief and transmission in online communities: An information cascade and group polarization perspective. *Inform Manage-Amster* (2017).

[4] Huo L, Wang L, Song G (2017). Global stability of a two-mediums rumor spreading model with media coverage. Phys. A..

[5] Zhao, L., Wang, J. & Huang, R. Immunization against the Spread of Rumors in Homogenous Networks. *Plos One* **10** (2015). 10.1371/journal.pone.0124978 PMC4416730 25933430

[6] Daley DJ, Kendall DG (1964). Epidemics + rumours. Nature.

[7] Moreno, Y., Nekovee, M. & Pacheco, A. F. Dynamics of rumor spreading in complex networks. *Phys Rev E* **69** (2004). 10.1103/PhysRevE.69.066130 15244690

[8] Nekovee M, Moreno Y, Bianconi G, Marsili M (2007). Theory of rumour spreading in complex social networks. Phys. A..

[9] Zhao L (2013). A rumor spreading model with variable forgetting rate. Phys. A..

[10] Zhao L (2011). Rumor spreading model with consideration of forgetting mechanism: A case of online blogging Live. Journal. Phys. A..

[11] Zhao L, Cui H, Qiu X, Wang X, Wang J (2013). SIR rumor spreading model in the new media age. Phys. A..

[12] Zhao L, Qiu X, Wang X, Wang J (2013). Rumor spreading model considering forgetting and remembering mechanisms in inhomogeneous networks. Phys. A.

[13] Zhao L (2012). SIHR rumor spreading model in social networks. Phys. A..

[14] Wang Y, Yang X, Han Y, Wang X (2013). Rumor Spreading Model with Trust Mechanism in Complex Social Networks. Commun. Theor. Phys..

[15] Zan Y (2018). DSIR double-rumors spreading model in complex networks. Chaos Soliton Fract..

[16] Zhang Y, Zhu J (2018). Stability analysis ofI2S2R rumor spreading model in complex networks. Phys. A..

[17] Wang J, Zhao L, Huang R (2014). 2SI2R rumor spreading model in homogeneous networks. Phys. A..

[18] Zan Y, Wu J, Li P, Yu Q (2014). SICR rumor spreading model in complex networks: Counterattack and self-resistance. Phys. A..

[19] Zhang Y, Su Y, Li W, Liu H (2018). Rumor and authoritative information propagation model considering super spreading in complex social networks. Phys. A..

[20] Huo L, Ma C (2017). Dynamical analysis of rumor spreading model with impulse vaccination and time delay. Phys. A..

[21] Xia L, Jiang G, Song B, Song Y (2015). Rumor spreading model considering hesitating mechanism in complex social networks. Phys. A..

[22] Hu Y, Pan Q, Hou W, He M (2018). Rumor spreading model with the different attitudes towards rumors. Phys. A..

[23] Zhao Z, Liu Y, Wang K (2016). An analysis of rumor propagation based on propagation force. Phys. A..

[24] Ma J, Li D, Tian Z (2016). Rumor spreading in online social networks by considering the bipolar social reinforcement. Phys. A..

[25] Sahafizadeh E, Tork Ladani B (2018). The impact of group propagation on rumor spreading in mobile social networks. Phys. A..

[26] Afassinou K (2014). Analysis of the impact of education rate on the rumor spreading mechanism. Phys. A..

[27] Wang, Y. & Wang, J. SIR rumor spreading model considering the effect of difference in nodes’ identification capabilities. *Int J. Mod Phys C* **28** (2017).

[28] Ma J, Zhu H (2018). Rumor diffusion in heterogeneous networks by considering the individuals’ subjective judgment and diverse characteristics. Phys. A..

[29] Li D, Ma J (2017). How the government’s punishment and individual’s sensitivity affect the rumor spreading in online social networks. Phys. A..

[30] Cheng, J., Liu, Y., Shen, B. & Yuan, W., An epidemic model of rumor diffusion in online social networks. *Eur Phys J. B* **86** (2013).

[31] Xia, L., Jiang, G., Song, B. & Zhu, G. In *Ninth International Conference on P2P, Parallel, Grid, Cloud and Internet Computing*, edited by F. Xhafa *et al*., pp. 161 (2014).

[32] Castillo, C., Mendoza, M. & Poblete, B. In *Proceedings of the 20th International Conference on World Wide Web*, pp. 675 (2011).

[33] Gou, L., Mahmud, J., Haber, E. & Zhou, M. In *Companion Publication of the 2013 International Conference on Intelligent User Interfaces Companion*, pp. 45 (2013).

[34] Longying, H. & Jingwei, D. Empirical Analysis of Micro-blog Users’ Forwarding Intention. *China soft science* 175 (2015).

[35] van den Driessche P, Watmough J (2002). Reproduction numbers and sub-threshold endemic equilibria for compartmental models of disease transmission. Math. Biosci..

[36] Askarizadeh M, Tork Ladani B, Manshaei MH (2019). An evolutionary game model for analysis of rumor propagation and control in social networks. Phys. A: Stat. Mech. its Appl..

